# Supplementary material for: Oncologic Benefits of Neoadjuvant Treatment versus Upfront Surgery in Borderline Resectable Pancreatic Cancer: A Systematic Review and Meta-Analysis
Source: Cancers (Basel). 2022 Sep 7;14(18):4360. doi: 10.3390/cancers14184360 (PMC9497278; doi:10.3390/cancers14184360)

Supplementary Figure S1. Risk of bias summary using the Cochrane Collaboration's tool for the randomized controlled trial (A) and the Risk of Bias Assessment Tool for Nonrandomized Studies (RoBANS) (B).

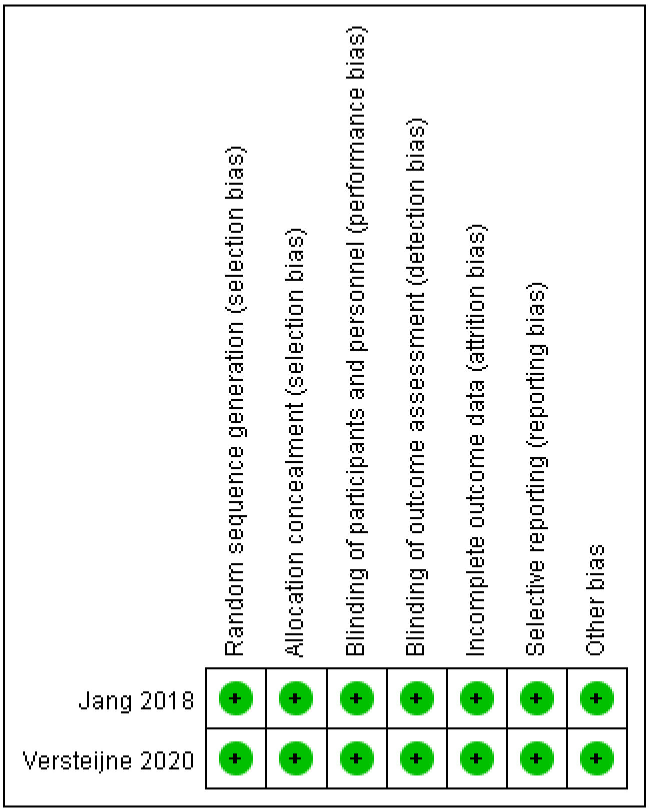

(A)

(B)

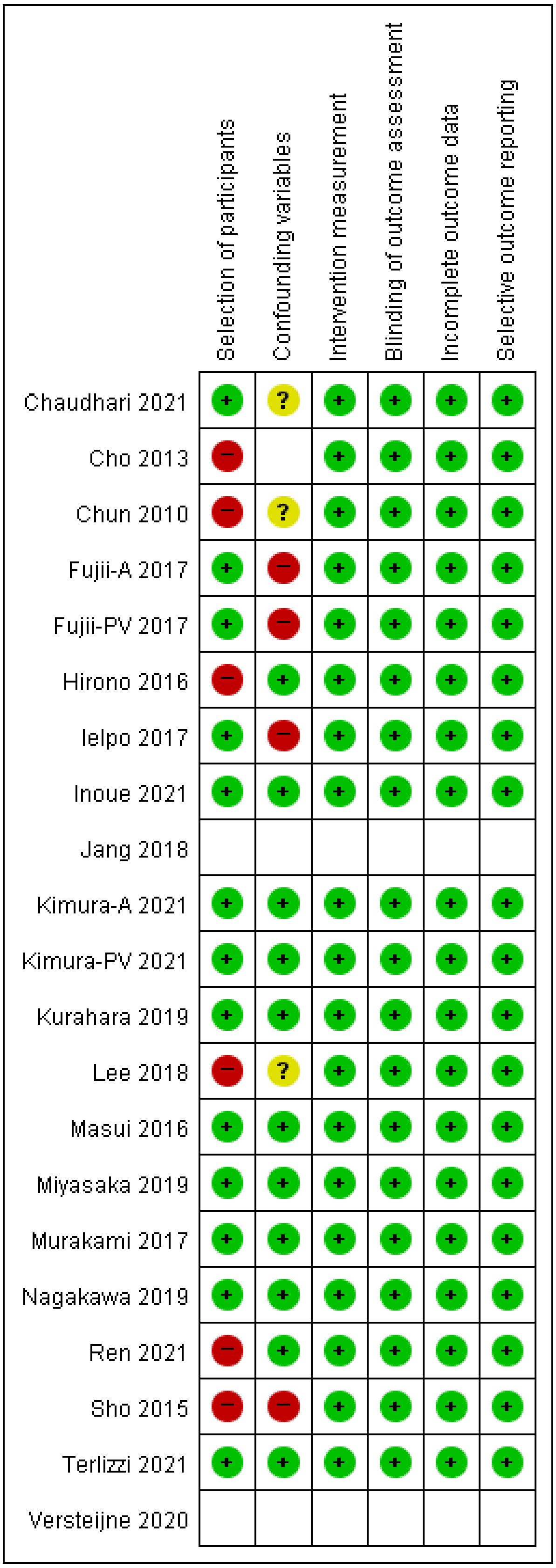
Selection of participants
Confounding variables
Intervention measurement
Blinding of outcome assessment
Incomplete outcome data
Selective outcome reporting
Chaudhari 2021
Cho 2013
Chun 2010
Fujii-A 2017
Fujii-PV 2017
Hirono 2016
Ielpo 2017
Inoue 2021
Jang 2018
Kimura-A 2021
Kimura-PV 2021
Kurahara 2019
Lee 2018
Masui 2016
Miyasaka 2019
Murakami 2017
Nagakawa 2019
Ren 2021
Sho 2015
Terlizzi 2021
Versteijne 2020

Supplementary Figure S2. Overall survival of NAT versus UFS according to NAT protocol (chemotherapy only and chemoradiotherapy).

Study or Subgroup	log[Hazard Ratio]	SE	Weight	Hazard Ratio IV, Fixed, 95% CI	Year
**1.1.1 Chemoradiotherapy**					
Fujii-A 2017	-0.23	0.13	18.2%	0.79 [0.62, 1.03]	2017
Fujii-PV 2017	-0.65	0.18	9.5%	0.52 [0.37, 0.74]	2017
Jang 2018	-0.67	0.31	3.2%	0.51 [0.28, 0.94]	2018
Kurahara 2019	-0.01	0.21	7.0%	0.99 [0.66, 1.49]	2019
Nagakawa 2019	-0.22	0.1	30.7%	0.80 [0.66, 0.98]	2019
Kimura-A 2021	-1.02	0.28	3.9%	0.36 [0.21, 0.62]	2021
Kimura-PV 2021	-0.96	0.35	2.5%	0.38 [0.19, 0.76]	2021
Terlizzi 2021	-0.61	0.37	2.2%	0.54 [0.26, 1.12]	2021
**Subtotal (95% CI)**			**77.2%**	**0.70 [0.62, 0.80]**	
Heterogeneity: Chi² = 18.26, df = 7 (P = 0.01); I² = 62%					
Test for overall effect: Z = 5.56 (P < 0.00001)					
**1.1.2 Chemotherapy only**					
Masui 2016	-0.24	0.58	0.9%	0.79 [0.25, 2.45]	2016
Murakami 2017	-0.73	0.25	4.9%	0.48 [0.30, 0.79]	2017
Miyasaka 2019	-1.02	0.38	2.1%	0.36 [0.17, 0.76]	2019
Versteijne 2020	-0.48	0.22	6.3%	0.62 [0.40, 0.95]	2020
Inoue 2021	-0.4	0.19	8.5%	0.67 [0.46, 0.97]	2021
**Subtotal (95% CI)**			**22.8%**	**0.58 [0.46, 0.73]**	
Heterogeneity: Chi² = 3.06, df = 4 (P = 0.55); I² = 0%					
Test for overall effect: Z = 4.69 (P < 0.00001)					
**Total (95% CI)**			**100.0%**	**0.67 [0.60, 0.75]**	
Heterogeneity: Chi² = 23.48, df = 12 (P = 0.02); I² = 49%					
Test for overall effect: Z = 7.13 (P < 0.00001)					
Test for subgroup differences: Chi² = 2.16, df = 1 (P = 0.14), I² = 53.6%					

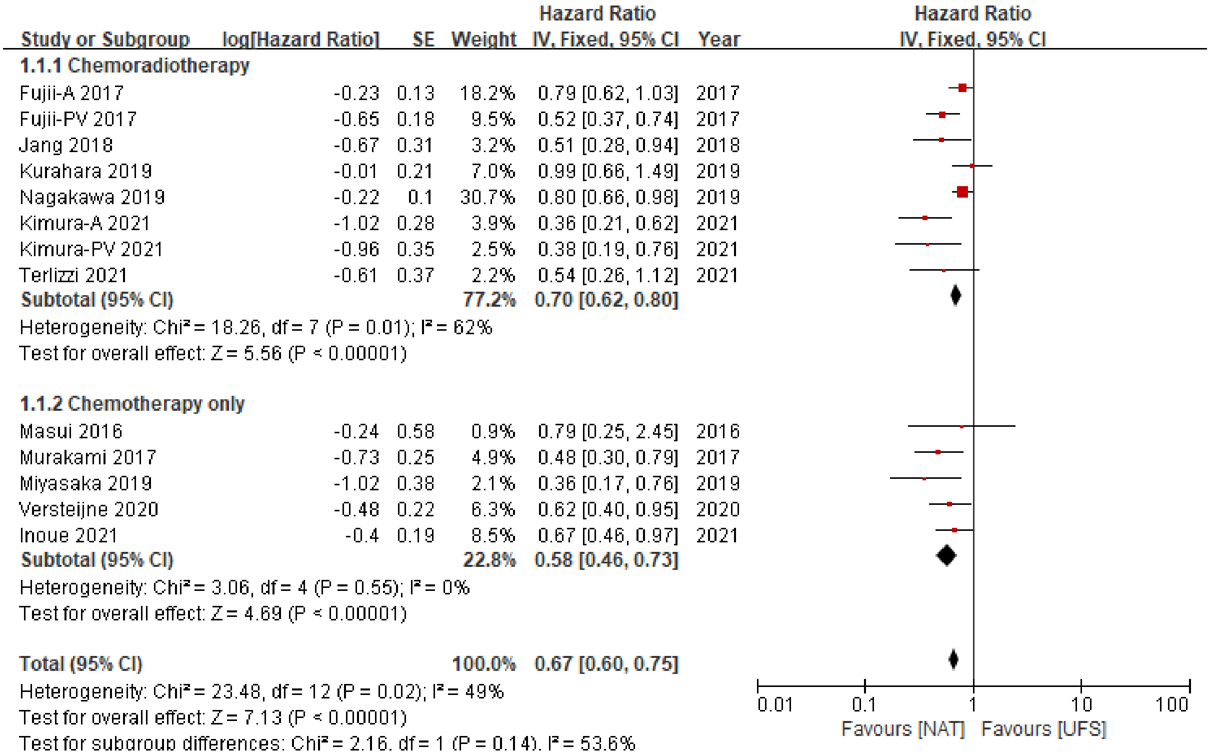

Supplementary Figure S3. Funnel plots for publication bias in comparison of survival outcome in ITT analysis.

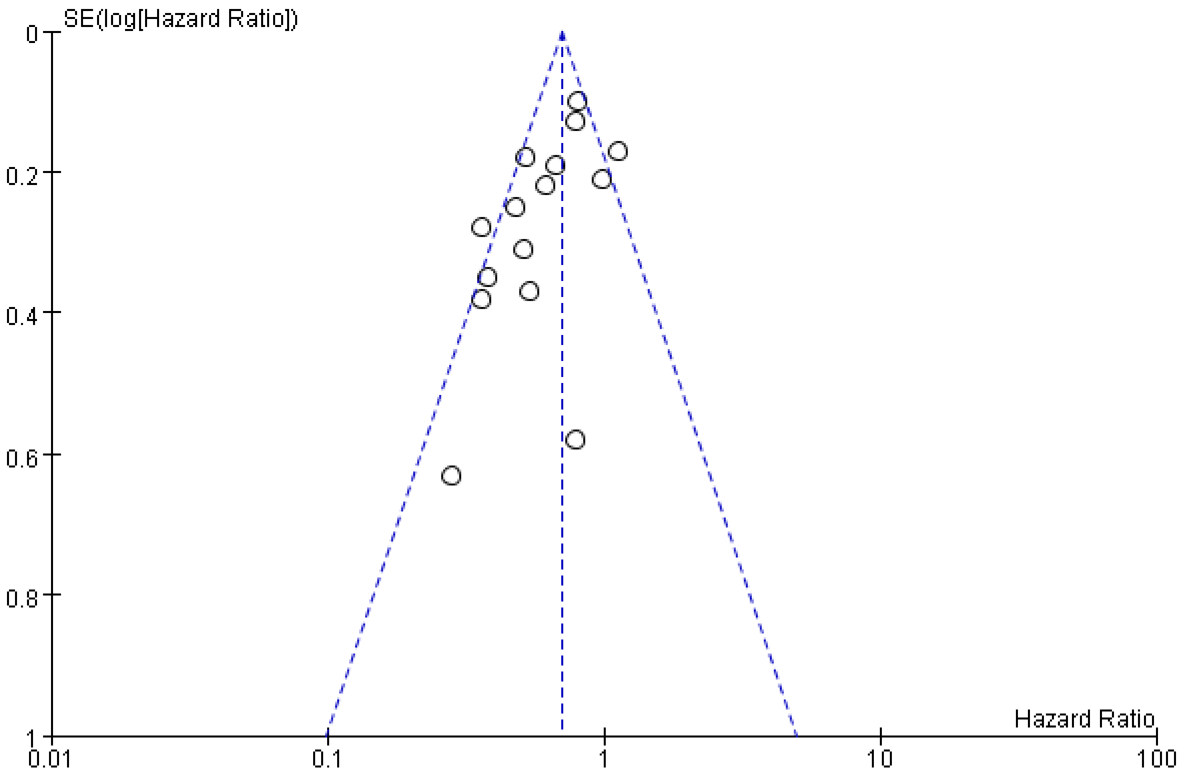

Supplement: Supplementary file 1 [file cancers-14-04360-s001.zip › cancers-1880288-supplementary.pdf]
